# Beneficial Contribution to Glucose Homeostasis by an Agro-Food Waste Product Rich in Abscisic Acid: Results from a Randomized Controlled Trial

**DOI:** 10.3390/foods11172637

**Published:** 2022-08-31

**Authors:** Elisabetta Schiano, Maria Maisto, Vincenzo Piccolo, Ettore Novellino, Giuseppe Annunziata, Roberto Ciampaglia, Camilla Montesano, Martina Croce, Giuseppe Caruso, Fortuna Iannuzzo, Vincenzo Summa, Gian Carlo Tenore

**Affiliations:** 1 Department of Pharmacy, University of Naples Federico II, via Domenico Montesano 59, 80131 Naples, Italy; 2 Department of Medicine and Surgery, Catholic University of the Sacred Heart, 00168 Rome, Italy; 3 Chemistry Department, University of Rome “Sapienza”, 00185 Rome, Italy; 4 Department of Emergency, Hospital Cardarelli, via Antonio Cardarelli, 80131 Naples, Italy

**Keywords:** abscisic acid, waste fruit products, nutraceutical, type 2 diabetes mellitus, glucose homeostasis

## Abstract

The control of glucose homeostasis represents the primary goal for the prevention and management of diabetes and prediabetes. In recent decades, the hypoglycemic hormone abscisic acid (ABA) has attracted considerable interest in the scientific literature. In this regard, the high ABA concentration in immature fruits led us to consider these food matrices as candidates for diabetes control. Therefore, the beneficial efficacy of a nutraceutical formulation based on thinned nectarines (TNs) rich in ABA was tested through a three-month, three-arm, parallel-group, randomized controlled trial (RCT) conducted on sixty-one patients with type 2 diabetes (T2D). After 3 months, both the treatments with low doses of TN (500 mg 3 times/day) and high doses of TN (750 mg 3 times/day) showed a significant reduction in glycemic parameters compared to baseline. Treatment with low doses of TN showed a greater insulin-sparing effect (fasting plasma insulin, FPI: −29.2%, *p* < 0.05 vs. baseline) compared to the high-dose group (FPI: −16.5%, *p* < 0.05 vs. baseline). Moreover, a significant correlation between glycemia and ABA plasmatic levels was observed for both intervention groups at baseline and after 3 months. Overall, our data reasonably support TN as a promising and innovative nutraceutical product able to contribute to the management of glucose homeostasis.

## 1. Introduction

Diabetes is a metabolic disease that has reached epidemic proportions; the most recent edition of the IDF Diabetes Atlas shows that in 2021, 10.5% of adults were affected by diabetes and that this percentage will further increase, along with 12% of global health expenditure destined to treat this condition [1]. Type 2 diabetes mellitus (T2DM) is the most prevalent form, affecting ~90–95% of diabetic subjects, and is characterized by decreased β-cell insulin secretion and/or insulin resistance, resulting in chronic hyperglycemia (elevated blood glucose levels) [1]. The application of therapeutic strategies aimed to contrast hyperglycemia is essential, especially in order to avoid diabetes-related microvascular and macrovascular complications [2]. Several trials indicated a wide range of approaches, such as modifications in lifestyle and pharmacological treatment [3], to ameliorate insulin sensitivity and β-cell function, contributing to the prevention of the onset and progression of diabetes [4]. Nevertheless, these interventions are often difficult to sustain due to side effects and poor adherence by patients [3,5], underlining the necessity to explore novel targeted strategies for the management of diabetes. In this regard, numerous studies have reported the effectiveness of some nutraceuticals in improving glycemic control and insulin sensitivity in subjects with altered glucose levels [6,7]. In the context of bioactive compounds with hypoglycemic potential, abscisic acid (ABA) has recently aroused considerable interest in the scientific literature as an endogenous hormone involved in the management of glucose homeostasis [8,9]. ABA is produced and released by pancreatic β-cells in response to high glucose concentrations in humans [10]. As a result, this molecule promotes stimulation of both glucose-dependent and glucose-independent insulin release [11], as well as stimulation of glucose uptake by upregulation of glucose transporter 4 (GLUT4) expression and translocation [10,12,13]. Specifically, these effects are mediated by the ABA interaction with its specific receptor, lanthionine synthetase C–like protein 2 (LANCL2) [14]. However, recent findings demonstrate that in vivo administration low doses of ABA may lead to improved glucose tolerance in association with an ameliorated insulin profile [15]. Overall, this evidence clarifies the role played by this hormone as a key factor in the control of diabetic pathology.

Despite the large number of studies focusing on ABA as an endogenous hormone [9,11], no food byproducts naturally rich in this bioactive compound have been proposed to date. Our scientific interest in thinned unripe fruits arose from the observation that ABA is not only produced by humans after glucose stimulation [10] but that it also represents a historically known phytohormone, the content of which reaches the highest concentration in a specific stage of immaturity in plants [16,17]. During this phase, crops may be subjected to fruit thinning, a typical agronomical practice carried out to improve fruit quality and size in harvest management. In this scenario, the large amount of unripe fruits discarded every year due to this process [18,19] represents an innovative and high-value sources of abscisic acid, in line with the concepts of food waste revaluation and environmental sustainability [19]. In support of this consideration, an impaired release of ABA was reported after a glucose load in patients with T2DM or gestational diabetes [20], further highlighting the potential of thinning byproducts as supplements of this hypoglycemic hormone. In this context, a previous HPLC-DAD screening performed by our research group identified thinned nectarines (TN) as the richest matrices of ABA among various thinning-derived waste products (i.e., apples, plums, pears, peaches, and nectarines) [21]. Moreover, the above-mentioned study demonstrated a significantly improved postprandial glycemic response in healthy humans after an acute administration of a TN-based formulation, in association with an insulin-sparing effect [21]. Subsequently, the beneficial properties of this byproduct were further investigated by qualitatively and quantitatively characterizing the polyphenolic composition through an HPLC-HESI-MS/MS analysis [22]. The resultant high concentration of polyphenolic compounds, together with the antioxidant and antidiabetic results obtained by in vitro assays, reasonably prompt us to test the clinical potential of TN supplementation with respect to glucose homeostasis.

In the light of these considerations, the aim of the current study was to evaluate the effects on glucose homeostasis management after supplementation with two different doses of a TN-based nutraceutical formulation through a randomized controlled trial (RCT) conducted on patients with type 2 diabetes (T2D). Moreover, the correlation between glycemia and ABA plasmatic levels was also evaluated on a subgroup of patients undergoing the clinical trial.

## 2. Materials and Methods

### 2.1. Study Population and Design

**Figure 1:**
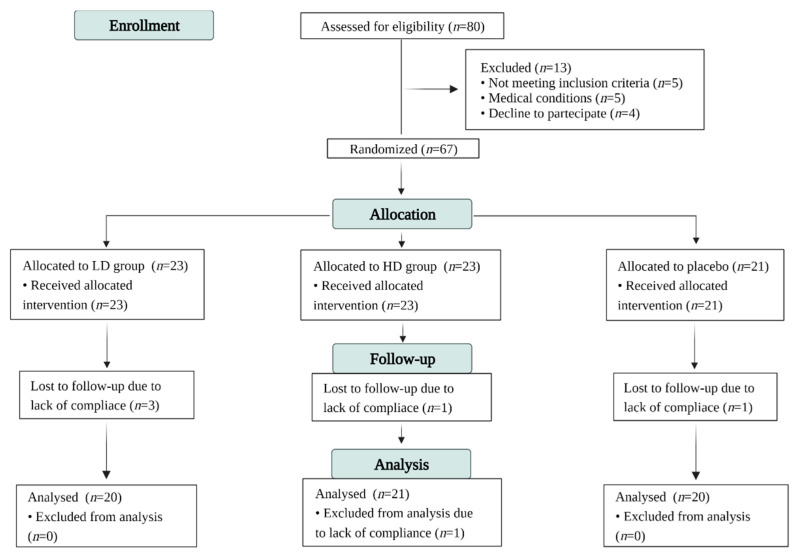
Consolidated Standards of Reporting Trials (CONSORT) flow diagram. HD, high-dose thinned nectarine group; LD, low-dose thinned nectarine group.

Study participants were recruited by the Samnium Medical Cooperative (Sant’Agata De’ Goti, Italy). Patients were enrolled in May 2019. The protocol, synoptic documents, and letters of intent of volunteers were submitted to the Scientific Ethics Committee of AO Rummo Hospital (Benevento, Italy) (Table S1). The study was approved by the committee (protocol no 28 of 15 May 2017) and conducted in accordance with the Declaration of Helsinki of 1964 (as revised in 2000). The sample size was calculated using G*Power 3.1.9.4 statistical analysis software to achieve 80% power. Patients aged 18–83 years with T2DM, as defined by the American Diabetes Association [23], for more than one year were eligible for enrolment. Figure 1 shows the flow of participants through the trial according to the criteria recommended in the CONSORT guidelines [24]. A total of 80 T2DM patients were initially invited to participate. Exclusion criteria were: type 1 diabetes mellitus (T1DM), smoking, renal disease, hepatic disease, heart disease, medications for chronic diseases, food supplement intake containing ABA or polyphenols, heavy physical exercise (>10 h/week), underweight (body mass index < 18.5 kg/m^2^), women suspected of being pregnant, women who hoped to become pregnant, pregnant women, breastfeeding, use of vitamin/mineral supplements 2 weeks before starting the study, birch pollen allergy, and donation of blood less than 3 months before the study. The occurrence of any of the above-reported exclusion criteria during the trial resulted in the immediate interruption of participation in the study. A total of eighty patients were initially enrolled in the trial. Thirteen patients did not qualify for the study as a result of inclusion/exclusion criteria. Ultimately, a total of sixty-seven patients received allocated intervention, sixty-one of whom completed the study. All the participants received oral and written information about the study before they signed their written consent.

The study was designed as a 12-week, monocentric, randomized, double-blind, placebo-controlled, 3-arm parallel-group trial. Patients were randomly allocated to three intervention groups: placebo (PL) group (500 mg of maltodextrins three times/day), low dose of TN (LD) group (500 mg of TN three times/day, lyophilized), or high dose of TN (HD) group (750 mg of TN three times/day, lyophilized). Both placebo and TN treatments were self-administered as tablets. TNs were harvested in June 2019 at the orchards of “Giaccio Frutta” society (Vitulazio, Caserta, Italy, 41°10′ N–14°13′ E) approximately 20–25 days after full bloom, coinciding with the stage of fruit thinning. Whole fruits were frozen at −80 °C, freeze-dried, and ground to obtain a homogenous powder, which represented the production batch used for the preparation of the tablets. Specifically, the large-scale production of tablets was accomplished by “La Sorgente del Benessere s.r.l.” (Fiuggi, Italy). Treatment compliance was assessed by counting the number of tablets returned at scheduled clinic visits. Throughout the study, we instructed patients to start the intervention on the day after they were given the appropriate treatments. All treatments were provided free of charge. Randomization was achieved by a drawing of envelopes containing randomization numbers. The random number list was created by an investigator with no clinical involvement in the trial. Patients, core laboratories, clinicians, and trial staff were blind to treatment allocation. Periodic and standardized telephone interviews were carried out by qualified personnel in order to verify and improve protocol compliance. Moreover, a secondary outcome of the study was represented by the measurement of plasmatic ABA (ABAp) levels through an LC-MS/MS analysis. This evaluation was only performed on a small subgroup of patients undergoing the clinical trial due to depletion of available samples (LD group at baseline, *n* = 9; LD group after 3 months, *n* = 6; HD group at baseline, *n* = 10; HD group after 3 months, *n* = 7).

### 2.2. Assessments

At the beginning of the study, all patients underwent a standardized physical examination, an assessment of medical history (up to five years before enrolment) and of vital signs (heart rate and blood pressure; laboratory examination; measurements of body height, body weight, and waist circumference (WC)), and evaluation of body mass index (BMI). BMI was calculated based on body height and weight. Blood samples were collected after 12 h of fasting at weeks 0, 4, 8, and 12 in 10-mL EDTA-coated tubes (Becton Dickinson, Plymouth, United Kingdom). Then, plasma was immediately isolated by centrifugation (20 min, 2.200 g, 4 °C). All samples were stored at −80 °C until analysis. Subjects were asked to abstain from alcohol consumption and practice strenuous physical activity 48 h prior to blood sampling. Plasma total cholesterol (TC), high-density lipoprotein cholesterol (HDL-C), low-density lipoprotein cholesterol (LDL-C), fasting plasma glucose (FPG), triglyceride (TG), aspartate aminotransferase (AST), alanine aminotransferase (ALT), and creatine levels were determined using commercially available kits from Diacron International (Grosseto, Italy). Analyses were performed on a Diacron International Free Carpe Diem analyzer. Glycated hemoglobin (HbA1c) was determined with a commercially available kit (InterMedical s.r.l, Italy). Fasting plasma insulin (FPI) was measured using an enzyme-linked immunosorbent (ELISA) assay commercial kit (InterMedical s.r.l, Grassobbio, Italy). Homeostatic model assessment of insulin resistance (HOMA index) was calculated with the following formula: FPG (mg/dL) times FPI (µUI/mL) divided by 22.5 [25].

### 2.3. Quantification of Plasmatic Abscisic Acid by LC-MS/MS Analysis

#### 2.3.1. Chemicals and Reagents

Methanol, acetonitrile, and other organic solvents were of chromatography grade from Sigma-Aldrich (Milano, Italy); water was purified using a milliQ system from Millipore (Billerica, MA, USA); and LC-MS-grade formic acid was purchased from Fluka (Milano, Italy). The internal standard (IS) 11-Nor-9-carboxy-Δ9-tetrahydrocannabinol (THC-COOH) was obtained from LGC standards (Sesto San Giovanni, MI, Italy) at a concentration of 0.1 mg mL^−1^ in methanol.

#### 2.3.2. Stock Solution Preparation

ABA stock solution was prepared in methanol at 1 mg mL^−1^ and stored at −20 °C. The stock solution was further diluted to obtain a working solution at 20 µg mL^−1^. internal standard working solution (IS-WS) was prepared by adding appropriate volumes of THC-COOH to 40 mL methanol:acetonitrile (50:50, *v*/*v*) in order to reach a final concentration of 10 ng mL^−1^. The solution was maintained at −20 °C.

#### 2.3.3. Sample Preparation

A volume of 50 µL of human plasma was mixed with 175 µL of IS-WS and 25 µL of methanol:acetonitrile (50:50, *v*/*v*). The mixture was vortexed for 1 min and centrifuged at 12,500× *g* for 10 min at 4 °C. The supernatant was collected, and 100 µL was injected into the LC-MS/MS system.

#### 2.3.4. LC-MS/MS Analysis

Analysis was performed by liquid chromatography tandem mass spectrometry (LC-MS/MS). The HPLC equipment consists of an LC AD system from AB Sciex (Toronto, ON, Canada). A 6500 Q-Trap mass spectrometer from AB Sciex (Toronto, ON, Canada) was used for detection. Chromatographic separation was achieved with an Acquity UPLC HSS-T3 1.8 µm column (2.1 × 50 mm ID) from Waters. The mobile phases were (B) ACN containing 0.01% formic acid and (A) water containing 0.01% formic acid at a flow rate of 0.5 mL min^−1^, which were entirely moved into the mass spectrometer source. The gradient elution was as follows: increase in the organic phase from 10 to 100% in 2.5 min; after 1.5 min of 100% B, the column was changed to conditions with 20% organic phase in 2.5 min to enable equilibration. ABA was detected in negative-ionization mode with a capillary voltage of −4500 V, nebulizer gas (air) at 35 psi, turbo gas (nitrogen) at 75 psi, and 450 °C. The other ion source parameters were set as follows: curtain gas (CUR), 30 psi; collision gas (CAD), medium; declustering potential (DP) −31 V; entrance potential (EP), −5 V. Instrument condition optimization was performed by direct infusion and manual tuning. Data collection and elaboration were performed by means of Analyst 1.7 software (AB-Sciex). Quantitative data were acquired using multireaction monitoring (MRM) mode. Two MRM transitions (precursor ion > fragment ion) were selected for the analytes. ABA transitions were *m*/*z* 263.1 > 153.0 and 263.1 > 204.0, collision energy (CE) was set at −16 and −28 V, and collision-cell exit potential (CXP) was set at −16 and −23 V for the two transitions, respectively. For the internal standard, transition was *m*/*z* 343.1 > 299.0, collision energy (CE) was set at −34 V, and collision-cell exit potential (CXP) was set at −33 V.

#### 2.3.5. Method Validation

The analytical method was validated according to the following parameters: linearity, precision, accuracy, limit of detection (LOD), and limit of quantification (LOQ). Calibration standard solutions and quality control samples (QCs) (*n* = 3 for each concentration) were prepared in a surrogate matrix (phosphate-buffered saline (PBS) solution) by spiking 25 µL of a standard mixture at an appropriate concentration to 50 µL of PBS and by adding 175 µL of methanol:acetonitrile (50:50, *v*/*v*). Calibrators were then treated similarly to animal samples. The calibration range was 0.4 to 20 ng mL^−1^, and the calibrators were prepared at eight levels of concentration. Precision and accuracy were evaluated at three level of concentration (0.5, 5, and 10 ng mL^−1^) and found to be within the acceptable limits. The limit of detection (LOD) was defined as the lowest concentration with a signal-to-noise (S/N) ratio greater than 3. Limit of quantification (LOQ) was defined as the concentration at which both precision (RSD%) and accuracy were less than 20%. LOQ was determined to be 0.4 ng mL^−1^, and LOD was 0.1 ng mL^−1^.

### 2.4. Statistics

Unless otherwise stated, all experimental results are expressed as the mean ± standard deviation (SD). Statistical analysis of data was performed by the Student’s t-test or Pearson’s correlation test. *P* values less than 0.05 were regarded as statistically significant. The degree of the linear relationship between two variables was evaluated using the Pearson product–moment correlation coefficient (R). Correlation coefficients (R) were calculated using GraphPad Prism 8.4.3.

## 3. Results

### 3.1. Study Sample

A total of eighty patients were initially enrolled in the trial; nineteen patients were lost due for various reasons. Ultimately, sixty-one participants (placebo group, *n* = 20; LD group, *n* = 20; HD group, *n* = 21) completed the 3 months of intervention. The baseline and 3-month general data and anthropometric parameters of patients are reported in Table 1. The groups were well-balanced for anthropometric and demographics factors. No changes were observed in BMI or circumference in the three treatment groups at the end of the clinical trial.

### 3.2. Glucometabolic Parameters

As shown in Table 2, a significant increase was observed in HDL-C values in both the treatment groups compared to baseline values (*p* < 0.05 vs. baseline), whereas no significant differences were observed in the placebo group. Additionally, a non-significant decrease and a significant decrease (*p* < 0.05 vs. baseline) in triglyceride (TG) levels were observed in the HD group and LD group, respectively. With respect to glycemic parameters, a significant decrease in FPG, FPI, HOMA-IR, and HbA1c was observed in the high-dose and low-dose TN treatment groups compared to the baseline values (*p* < 0.05 vs. baseline), whereas no significant differences were observed in the placebo group.

Although a significant decrease in glycemic parameters was observed in both TN treatment groups, treatment with low doses of TN showed a greater decrease in FPG values (−14.8%, *p* < 0.01 vs. baseline) compared to the high-dose TN group (−6.9%, *p* < 0.05 vs. baseline) and a greater insulin-sparing effect (FPI: −29.2%, *p* < 0.05 vs. baseline) compared to the high-dose TN group (FPI: −16.5%, *p* < 0.05 vs. baseline). Overall, as reported in Table 3, these results indicate a greater decrease in HOMA-IR values in the low-dose TN group (−41.6%, *p* < 0.05 vs. baseline) compared to the high-dose TN group (−22.2%, *p* < 0.05 vs. baseline).

### 3.3. Correlation Analysis of ABA Plasmatic Levels and Glycemia in Groups Treated with Low and High Doses of Thinned Nectarines

**Figure 2:**
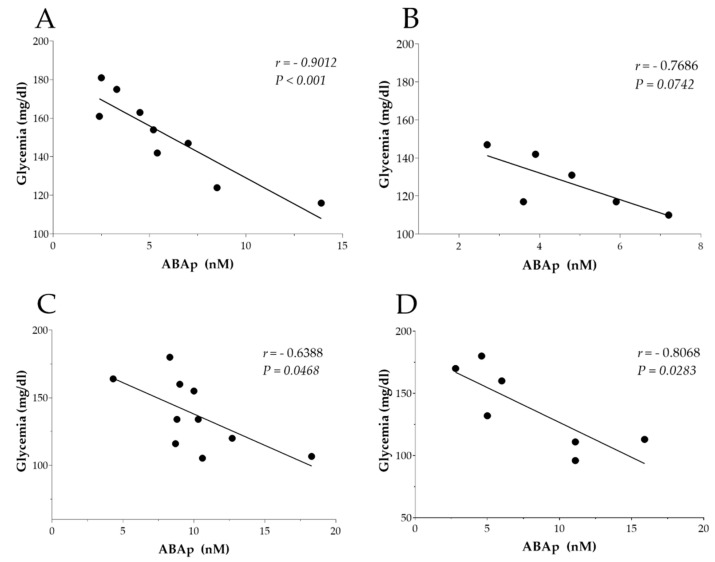
Correlation analysis data. Pearson correlation analysis was performed to evaluate the link between plasmatic ABA levels and glycemia in the following groups: (**A**) low-dose TN group (500 mg of TN three times/day) at baseline (T0), (**B**) low-dose TN group (500 mg of TN three times/day) after 90 days (T90), (**C**) high-dose TN group (750 mg of TN three times/day) at baseline (T0), and (**D**) high-dose TN group (750 mg of TN three times/day) after 90 days (T90). Abbreviations: ABA, abscisic acid; TN, thinned nectarines.

A secondary outcome of the present work included the investigation of the relationship between plasma ABA concentration (ABAp) and changes in plasma glucose levels in patients supplemented with LD or HD of TN at baseline and after 3 months. To this end, an LC-MS/MS analysis was performed to determine the ABAp levels, and the obtained data were correlated with the corresponding glycemic values in a small subgroup of patients (LD group at baseline, *n* = 9; LD group after 3 months, *n* = 6; HD group at baseline, *n* = 10; HD group after 3 months, *n* = 7). As shown in Figure 2, a negative correlation was observed for both groups treated with the TN-based nutraceutical product at baseline and after 3 months. Specifically, these correlations were statistically significant in the LD group at T0 (*r* = −0.9012, *p* < 0.001), in the HD group at T0 (*r* = −0.6388, *p* = 0.0468), and in the HD group at T90 (*r* = −0.8068, *p* = 0.0283), although a non-significant negative correlation was also observed in the LD group at T90 (*r* = −0.7686, *p* = 0.0742).

## 4. Discussion

Abscisic acid is widely described as a terpenoid phytohormone naturally present in fruits and vegetables [26], with a crucial role in controlling glucose homeostasis in humans [9]. This bioactive compound is currently and extensively being investigated for various therapeutic purposes, such as treatment of diabetes, cancer, ischemic retinopathy, and neurodegenerative diseases, due to its broad attributed biological activity spectrum [27,28,29]. The first aim of this study was to evaluate the potential beneficial effects on glucose homeostasis management after supplementation with two different doses of a TN-based nutraceutical product rich in ABA. The treatments were found to significantly reduce fasting glycemia in both intervention groups, with a greater decrease in FPG values in the LD group (−14.8%, *p* < 0.01, vs. baseline) compared to the HD group (−6.9%, *p <* 0.05 vs. baseline) after 3 months. A greater insulin -paring effect was also reported in patients treated with low doses of TN (FPI: −29.2%, *p* < 0.05 vs. baseline) compared to the HD group (FPI: −16.5%, *p* < 0.05 vs. baseline).

These results seem to be consistent with available scientific data involving the supplementation with ABA. Recent studies showed that the mechanism behind the hypoglycemic action of ABA supplemented in vivo at low doses (few micrograms/kg body weight) depends on its ability to upregulate the peripheral glucose transport rather than its stimulatory activity upon insulin release [15]. Although the reasons for this unexpected effect have not yet been fully elucidated, various hypotheses have been proposed. Based on these assumptions, the increased glucose uptake in GLUT4-expressing cells by ABA could exceed in extent and/or precede in time the stimulation of insulin release, or, in a not necessarily exclusive manner, these cells may be more responsive to the effect of ABA than β-pancreatic cells in vivo [8]. Therefore, low amounts of ABA may be suggested as a useful tool to ameliorate glucose tolerance in subjects with a deficiency of/or insulin resistance. In this regard, there is a growing consensus among the scientific community that the protracted stimulation of insulin release from β-pancreatic cells under conditions of chronic hyperglycemia may contribute to their eventual exhaustion [30]. Taking into account this evidence, antidiabetic molecules capable of decreasing glycemia without increasing insulinemia are highly desirable, as they could improve the survival and function of these cells. In this context, byproduct nectarines derived from fruit thinning may be considered valuable matrices for nutraceutical applications. In particular, the results presented herein may direct physicians toward novel treatments/interventions that could represent a useful alternative/support in clinical practice.

One of the objectives of the present work was the investigation of the relationship between plasma ABAp levels and changes in plasma glucose levels in patients undergoing the clinical trial at baseline and after 3 months. Although not always significant, a negative correlation was observed for both groups treated with the TN-based nutraceutical formulations at baseline and at the end of the trial. In agreement with the observed data, an impaired release of ABA was reported after a glucose load in patients with T2DM or gestational diabetes [20]. Another convincing indication that ABA could be involved in glycemia control is derived from the observation that nanomolar ABA stimulated glucose and insulin uptake in murine 3T3-L1 cells differentiated to adipocytes and in rat L6 myoblasts by upregulating GLUT-4 translocation [10]. Additionally, the insulin-independent hypoglycemic activity of ABA was suggested by recent ex vivo experiments in which ABA was demonstrated to stimulate the uptake of a fluorescent glucose analog by mouse skeletal muscle in the absence of insulin, with LANCL2 silencing reducing all observed effects [13]. These results were also confirmed in vivo in rats undergoing an oral glucose load, as ABA-induced glucose and storage in skeletal muscles were detected by micro-PET [13]. From a mechanistic point of view, the activation of AMPK could be responsible for these effects. More specifically, incubation with ABA was shown to increase phosphorylation of AMPK, leading to the phosphorylation and activation of PGC-1α in skeletal muscle. Eventually, the main consequence of this process could be represented by an increase in GLUT4 expression and glycogen accumulation [13,31]. AMPK also represents an upstream positive regulator of p38 MAPK [32], which has been attributed its ability to promote PPAR-γ phosphorylation on Ser122, therefore preventing PPAR-γ-induced inhibition of GLUT4 expression [13,33,34]. Altogether, these observations of the non-overlapping signaling pathways and the metabolic effects of ABA and insulin suggest a role of ABA as the first hormonal actor in the glycemic response.

With respect to the lipid profile, a significant increase in HDL-C values was observed in both the groups treated with TN compared to baseline, whereas no significant differences were observed in the placebo group. This unexpected result could be explained by the high content of polyphenolic compounds in our food matrix [22]. As reported in scientific studies, polyphenols can positively influence cholesterol metabolism, especially regarding the B2 class of dimeric procyanidins [35]. Moreover, the decrease in TG levels observed in the TN treatment groups may be justified both by the reported beneficial effects exerted by polyphenols on the management of hypertriglyceridemia [36] and by the positive response of plasma TG levels resulting from improved glycemic control, as described by scientific evidence [37]. In agreement with this hypothesis, our characterization of the TN polyphenolic profile performed by a validated HPLC-DAD method [38] highlights the high potential of this matrix in terms of polyphenol content [22]. In this sense, increasing evidence suggests a pivotal role played by oxidative stress in the pathogenesis of both types of DM [39,40] and the related beneficial role exerted by polyphenolic compounds [41,42]. Specifically, high levels of free radicals and the simultaneous decrease in the antioxidant defense mechanisms can lead to damage of biological structures, with a resulting increased risk of diabetes-related complications [41]. Correspondingly, persistent hyperglycemia is known as one of the main causes of oxidative stress, suggesting a direct cause-and-effect relationship between hyperglycemia and oxidative stress [43]. Based on these considerations, the concurrent presence of bioactive molecules with antidiabetic and antioxidant potential in TN further suggests its synergistic efficacy for the management of diabetic pathology.

### Strengths and Limitations

One of the strengths of the clinical trial herein presented is the originality of the study design, which involves the administration of an agricultural waste product as a source of bioactive compounds with hypoglycemic potential. This is in line with the current worldwide trend of re-evaluating such byproducts for purposes of environmental sustainability and circular economy. Secondly, the study population represented by patients with T2DM who maintained their usual daily diets allowed us to evaluate treatment effects in a real-world setting. Moreover, the two tested doses of TN in the intervention groups contributed to evaluation of a dose-response relationship, helping to identify the adequate dose of the tested food supplement. In this context, the reported results can inform physicians about an innovative intervention that could represent a valuable alternative in clinical practice. Finally, although the ABAp concentration was only measured in a limited number of samples, the analysis allowed us to highlight an existing correlation between the circulating amount of ABA and the observed effects on glycemic levels. These preliminary results strongly encourage us to consider measuring ABAp levels as a primary outcome in future studies with an increased number of patients. The main limitations of our study include the choice of exclusively white race, the short-term assessment for the treatment of a chronic condition, and the wide-ranging age of participants due to availability during enrollment. Therefore, randomized, placebo-controlled studies with larger numbers of participants are warranted to further confirm our observations.

## 5. Conclusions

In conclusion, the present study indicates that the investigated TN-based nutraceutical product may significantly and clinically reduce glucose levels, in association with an insulin-saving effect. Overall, these results reasonably support TN as an innovative and promising nutraceutical formulation able to contribute to the management of glucose homeostasis. According to available scientific data, the observed beneficial effects may be attributed to the role of ABA in TN, mainly relating to the insulin-sparing mechanism of action. Moreover, the negative correlations observed between glycemia and ABA plasmatic levels in patients treated with TN further support the observed beneficial results. Undoubtedly, the present work needs to be supported by further investigations to confirm the reported results.

## Figures and Tables

**Table 1:** Baseline and 3-month general data and anthropometric parameters of randomized subjects in the placebo, high-dose, and low-dose thinned nectarine groups.

Parameter	Low Doses of TN		High Doses of TN		Placebo	
	Baseline	12 Weeks	Baseline	12 Weeks	Baseline	12 Weeks
Patients (n)	20	20	21	21	20	20
M/F	11/9	-	12/9	-	13/7	-
Age (years)	63 ± 6.5	-	64 ± 7.8	-	64 ± 5.9	-
Height (m)	1.69 ± 0.08	-	1.66 ± 0.09	-	1.68 ± 0.07	-
Weight (kg)	83.4 ± 7.9	83.2 ± 6.8	86.1 ± 6.2	85.9 ± 4.2	85.4 ± 4.5	85.3 ± 4.4
BMI (kg/m^2^)	29.2 ± 4.1	29.1 ± 4.1	31.6 ± 4.5	31.6 ± 4.5	30.5 ± 4.3	30.5 ± 4.2
WC (cm)	99.0 ± 8.5	98.9 ± 8.6	102.8 ± 6.8	102.7 ± 6.9	99.5 ± 7.8	99.5 ± 7.9

Data are expressed as mean ± standard deviation. BMI: body mass index; F: female subject; M: male subjects; TN: thinned nectarine; WC: waist circumference.

**Table 2:** Baseline and 3-month biochemical parameters of patients in the placebo, high-dose and low-dose of thinned nectarine groups.

Parameter	Low Doses of TN (*n* = 20)		High Doses of TN (*n* = 21)		Placebo (*n* = 20)	
	Baseline	3 Months	Baseline	3 Months	Baseline	3 Months
FPG (mg/dL)	151.4 ± 28.6	129.9 ± 12.7 **	147.2 ± 36.8	137.1 ± 26.9 *	155.1 ± 31.5	154.6 ± 30.6
FPI (µU/mL)	12.0 ± 9.1	8.5 ± 4.0 *	15.22 ± 7.6	12.77 ± 6.6 *	15.9 ± 6.7	15.4 ± 6.9
HbA1c (%)	8.0 ± 0.9	6.9 ± 0.7 ***	7.8 ± 0.6	6.9 ± 0.4 ***	7.7 ± 0.5	7.7 ± 0.3
HOMA-IR	4.7 ± 4.0	2.7 ± 1.2 *	5.6 ± 3.3	4.4 ± 2.6 *	5.8 ± 3.2	5.8 ± 3.2
TC (mg/dL)	146.4 ± 36.1	152.8 ± 30.9	164.2 ± 27.0	167.5 ± 26.5	150.5 ± 28.9	151.6 ± 29.8
LDL-C (mg/dL)	83.4 ± 28.8	83.7 ± 26.5	109.5 ± 19.8	108.8 ± 31	84.5 ± 35.9	84.6 ± 34.8
HDL-C (mg/dL)	36.2 ± 8.1	39.0 ± 8.3 *	34.2 ± 8.7	39.0 ± 7.9 *	36.7 ± 7.5	36.6 ± 7.2
TG (mg/dL)	134.3 ± 42.1	100.6 ± 22.1 **	102.7 ± 27.5	98.5 ± 28.5	130.5 ± 32.1	133.6 ± 33.2
AST (UI/L)	25.7 ± 8.2	21.7 ± 6.2	21.5 ± 6.3	21.1 ± 5.6	24.5 ± 4.6	24.3 ± 4.8
ALT (UI/L)	18.9 ± 6.3	20.0 ± 6.3	19.3 ± 4.7	18.4 ± 3.6	19.8 ± 5.6	19.9 ± 5.5
Cre (mg/dL)	0.9 ± 0.2	0.9 ± 0.1	1.2 ± 0.1	1.1 ± 0.2	0.9 ± 0.3	0.9 ± 0.1

Data are expressed as mean ± standard deviation. Statistical significance was calculated by Student’s t-test analysis; * *p* < 0.05; ** *p* < 0.01; *** *p* < 0.001 vs. baseline. ALT: alanine aminotransferase; AST: aspartate aminotransferase; Cre: creatinine; FPG: fasting plasma glucose; FPI: fasting plasma insulin; HbA1c: glycated hemoglobin; HDL-C: high-density lipoprotein cholesterol; HOMA-IR: homeostatic model assessment of insulin resistance; LDL-C: low-density lipoprotein cholesterol; TC: total cholesterol; TG: triglycerides; TN: thinned nectarine.

**Table 3:** Effects of low and high doses of thinned nectarines on glycemic parameters at baseline and after 3 months.

	Fasting Plasma Glucose		Fasting Plasma Insulin		HOMA-IR	
HD Treatment Group	Baseline	3 Months	Baseline	3 Months	Baseline	3 Months
Mean ± SD	147.2 ± 36.8	137.1 ± 26.9	15.2 ± 7.6	12.7 ± 6.6	5.6 ± 3.3	4.4 ± 2.6
% Variation		**−6.9%**		**−16.5%**		**−22.2%**
*p*-value		0.0472		0.0345		0.0482
**LD Treatment Group**						
Mean ± SD	151.4 ± 28.6	129.9 ± 12.7	12.0 ± 9.1	8.5 ± 4.0	4.7 ± 4.0	2.5 ± 1.2
% Variation		**−14.8%**		**−29.2%**		**−41.6%**
*p*-value		0.0035		0.0404		0.0230

HD, high-dose thinned nectarine group; LD, low-dose thinned nectarine group; SD, standard deviation; HOMA-IR: homeostatic model assessment of insulin resistance.

## Data Availability

Data is contained within the article.

## Supplementary Materials

The following supporting information can be downloaded at: https://www.mdpi.com/article/10.3390/foods11172637/s1, Table S1: CONSORT 2010 checklist of information to include when reporting a randomised trial.

## References

[1] Sun H., Saeedi P., Karuranga S., Pinkepank M., Ogurtsova K., Duncan B.B., Stein C., Basit A., Chan J.C.N., Mbanya J.C. (2022). IDF Diabetes Atlas: Global, regional and country-level diabetes prevalence estimates for 2021 and projections for 2045. Diabetes Res. Clin. Pract..

[2] Doumas M., Imprialos K., Stavropoulos K., Athyros V.G. (2020). Pharmacological Management of Type 2 Diabetes Complications. Curr. Vasc. Pharmacol..

[3] Krass I., Schieback P., Dhippayom T. (2015). Adherence to diabetes medication: A systematic review. Diabet. Med..

[4] Marium R., Khan M., Jia Z., Chua Y., Tan J.C., Yang Y., Liao Z., Zhao Y. (2019). medicina From Pre-Diabetes to Diabetes: Diagnosis, Treatments and Translational Research. Medicina.

[5] Tan S.Y., Mei Wong J.L., Sim Y.J., Wong S.S., Mohamed Elhassan S.A., Tan S.H., Ling Lim G.P., Rong Tay N.W., Annan N.C., Bhattamisra S.K. (2019). Type 1 and 2 diabetes mellitus: A review on current treatment approach and gene therapy as potential intervention. Diabetes Metab. Syndr. Clin. Res. Rev..

[6] Li R., Zhang Y., Rasool S., Geetha T., Babu J.R. (2019). Review Article Effects and Underlying Mechanisms of Bioactive Compounds on Type 2 Diabetes Mellitus and Alzheimer’s Disease. Oxidative Med. Cell. Longev..

[7] Fernandes I., Oliveira J., Pinho A., Carvalho E. (2022). The Role of Nutraceutical Containing Polyphenols in Diabetes Prevention. Metabolites.

[8] Zocchi E., Hontecillas R., Leber A., Einerhand A., Carbo A., Bruzzone S., Tubau-Juni N., Philipson N., Zoccoli-Rodriguez V., Sturla L. (2017). Abscisic Acid: A Novel Nutraceutical for Glycemic Control. Front. Nutr..

[9] Magnone M., Sturla L., Guida L., Spinelli S., Begani G., Bruzzone S., Fresia C., Zocchi E. (2020). Abscisic acid: A conserved hormone in plants and humans and a promising aid to combat prediabetes and the metabolic syndrome. Nutrients.

[10] Bruzzone S., Ameri P., Briatore L., Mannino E., Basile G., Andraghetti G., Grozio A., Magnone M., Guida L., Scarfì S. (2012). The plant hormone abscisic acid increases in human plasma after hyperglycemia and stimulates glucose consumption by adipocytes and myoblasts; The plant hormone abscisic acid increases in human plasma after hyperglycemia and stimulates glucose consumption by adipocytes and myoblasts. FASEB J. Res. Commun..

[11] Bruzzone S., Bodrato N., Usai C., Guida L., Moreschi I., Nano R., Antonioli B., Fruscione F., Magnone M., Scarfì S. (2008). Abscisic acid is an endogenous stimulator of insulin release from human pancreatic islets with cyclic ADP ribose as second messenger. J. Biol. Chem..

[12] Sturla L., Mannino E., Scarfì S., Bruzzone S., Magnone M., Sociali G., Booz V., Guida L., Vigliarolo T., Fresia C. (2017). Abscisic acid enhances glucose disposal and induces brown fat activity in adipocytes in vitro and in vivo. Biochim. Biophys. Acta-Mol. Cell Biol. Lipids.

[13] Magnone M., Emionite L., Guida L., Vigliarolo T., Sturla L., Spinelli S., Buschiazzo A., Marini C., Sambuceti G., De Flora A. (2020). Insulin-independent stimulation of skeletal muscle glucose uptake by low-dose abscisic acid via AMPK activation. Sci. Rep..

[14] Sturla L., Fresia C., Guida L., Bruzzone S., Scarfi S., Usai C., Fruscione F., Magnone M., Millo E., Basile G. (2009). LANCL2 is necessary for abscisic acid binding and signaling in human granulocytes and in rat insulinoma cells. J. Biol. Chem..

[15] Magnone M., Ameri P., Salis A., Andraghetti G., Emionite L., Murialdo G., De Flora A., Zocchi E. (2015). Microgram amounts of abscisic acid in fruit extracts improve glucose tolerance and reduce insulinemia in rats and in humans. FASEB J..

[16] Leng P., Yuan B., Guo Y., Chen P. (2014). The role of abscisic acid in fruit ripening and responses to abiotic stress. J. Exp. Bot..

[17] Bai Y., Xiong C., Yin X., Ye T., Cai B., Song W., Feng Y. (2022). Screening and Identi fi cation of Potential Abscisic Acid Catabolites by Chemical Labeling-Assisted Ultrahigh-Performance Liquid Chromatography Coupled with High-Resolution Mass Spectrometry. J. Agric. Food Chem..

[18] Raak N., Symmank C., Zahn S., Aschemann-Witzel J., Rohm H. (2017). Processing- and product-related causes for food waste and implications for the food supply chain. Waste Manag..

[19] Mengyuan W., Haoli W., Tingting M., Qian G., Yulin F., Xiangyu S. (2021). Comprehensive Utilization of Thinned Unripe Fruits from Horticultural Crops. Foods.

[20] Ameri P., Bruzzone S., Mannino E., Sociali G., Andraghetti G., Salis A., Ponta M.L., Briatore L., Adami G.F., Ferraiolo A. (2015). Impaired increase of plasma abscisic acid in response to oral glucose load in type 2 diabetes and in gestational diabetes. PLoS ONE.

[21] Tenore G.C., Caruso D., D’avino M., Buonomo G., Caruso G., Ciampaglia R., Schiano E., Maisto M., Annunziata G., Novellino E. (2020). A pilot screening of agro-food waste products as sources of nutraceutical formulations to improve simulated postprandial glycaemia and insulinaemia in healthy subjects. Nutrients.

[22] Schiano E., Piccolo V., Novellino E., Maisto M., Iannuzzo F., Summa V., Tenore G.C. (2022). Thinned Nectarines, an Agro-Food Waste with Antidiabetic Potential: HPLC-HESI-MS/MS Phenolic Characterization and In Vitro Evaluation of Their Beneficial Activities. Foods.

[23] Gavin J.R., Alberti K.G.M.M., Davidson M.B., DeFronzo R.A., Drash A., Gabbe S.G., Genuth S., Harris M.I., Kahn R., Keen H. (1998). Report of the expert committee on the diagnosis and classification of diabetes mellitus. Diabetes Care.

[24] Calvert M., Blazeby J., Altman D.G., Revicki D.A., Moher D., Brundage M.D. (2013). Reporting of patient-reported outcomes in randomized trials: The CONSORT PRO extension. JAMA..

[25] Bonora E., Formentini G., Calcaterra F., Lombardi S., Marini F., Zenari L., Saggiani F., Poli M., Perbellini S., Raffaelli A. (2002). HOMA-estimated insulin resistance is an independent predictor of cardiovascular disease in type 2 diabetic subjects: Prospective data from the Verona Diabetes Complications Study. Diabetes Care.

[26] Brookbank B.P., Patel J., Gazzarrini S., Nambara E. (2021). Role of basal aba in plant growth and development. Genes.

[27] Sanchez-Perez A. (2020). Abscisic acid, a promising therapeutic molecule to prevent Alzheimer’s and neurodegenerative diseases. Neural Regen. Res..

[28] Baliño P., Gómez-Cadenas A., López-Malo D., Romero F.J., Muriach M. (2019). Is There A Role for Abscisic Acid, A Proven Anti-Inflammatory Agent, in the Treatment of Ischemic Retinopathies?. Antioxidants.

[29] Sakthivel P., Sharma N., Klahn P., Gereke M., Bruder D. (2016). Abscisic Acid: A Phytohormone and Mammalian Cytokine as Novel Pharmacon with Potential for Future Development into Clinical Applications. Curr. Med. Chem..

[30] Muoio D.M., Newgard C.B. (2008). Mechanisms of disease: Molecular and metabolic mechanisms of insulin resistance and β-cell failure in type 2 diabetes. Nat. Rev. Mol. Cell Biol..

[31] Leick L., Fentz J., Biensø R.S., Knudsen J.G., Jeppesen J., Kiens B., Wojtaszewski J.F.P., Pilegaard H. (2010). PGC-1α is required for AICAR-induced expression of GLUT4 and mitochondrial proteins in mouse skeletal muscle. Am. J. Physiol.-Endocrinol. Metab..

[32] Xi X., Han J., Zhang J.Z. (2001). Stimulation of Glucose Transport by AMP-activated Protein Kinase via Activation of p38 Mitogen-activated Protein Kinase. J. Biol. Chem..

[33] Armoni M., Kritz N., Harel C., Bar-Yoseph F., Chen H., Quon M.J., Karnieli E. (2003). Peroxisome proliferator-activated receptor-γ represses GLUT4 promoter activity in primary adipocytes, and rosiglitazone alleviates this effect. J. Biol. Chem..

[34] Magnone M., Spinelli S., Begani G., Guida L., Sturla L., Emionite L., Zocchi E. (2022). Abscisic Acid Improves Insulin Action on Glycemia in Insulin-Deficient Mouse Models of Type 1 Diabetes. Metabolites.

[35] Tenore G.C., Carotenuto A., Caruso D., Buonomo G., D’Avino M., Brancaccio D., Ciampaglia R., Maisto M., Schisano C., Novellino E. (2018). A nutraceutical formulation based on Annurca apple polyphenolic extract is effective on intestinal cholesterol absorption: A randomised, placebo-controlled, crossover study. PharmaNutrition.

[36] Schiano E., Annunziata G., Ciampaglia R., Iannuzzo F., Maisto M., Tenore G.C., Novellino E. (2020). Bioactive Compounds for the Management of Hypertriglyceridemia: Evidence From Clinical Trials and Putative Action Targets. Front. Nutr..

[37] Georgopoulos A., Margolis S., Bachorik P., Kwiterovich P.O. (1988). Effect of improved glycemic control on the response of plasma triglycerides to ingestion of a saturated fat load in normotriglyceridemic and hypertriglyceridemic diabetic subjects. Metabolism..

[38] Maisto M., Schiano E., Novellino E., Piccolo V., Iannuzzo F., Salviati E., Summa V., Annunziata G., Tenore G.C. (2022). Application of a Rapid and Simple Technological Process to Increase Levels and Bioccessibility of Free Phenolic Compounds in Annurca Apple Nutraceutical Product. Foods.

[39] Blesia V., Patel V.B., Al-Obaidi H., Renshaw D., Zariwala M.G. (2021). Excessive Iron Induces Oxidative Stress Promoting Cellular Perturbations and Insulin Secretory Dysfunction in MIN6 Beta Cells. Cells.

[40] Ježek P., Hlavatá L. (2005). Mitochondria in homeostasis of reactive oxygen species in cell, tissues, and organism. Int. J. Biochem. Cell Biol..

[41] Maritim A.C., Sanders R.A., Watkins J.B. (2003). Diabetes, oxidative stress, and antioxidants: A review. J. Biochem. Mol. Toxicol..

[42] Lorinczova H.T., Begum G., Temouri L., Renshaw D., Zariwala M.G. (2022). Co-Administration of Iron and Bioavailable Curcumin Reduces Levels of Systemic Markers of Inflammation and Oxidative Stress in a Placebo-Controlled Randomised Study. Nutrients.

[43] Tangvarasittichai S. (2015). Oxidative stress, insulin resistance, dyslipidemia and type 2 diabetes mellitus. World J. Diabetes.

